# Identifying functional subtypes of IgA nephropathy based on three machine learning algorithms and WGCNA

**DOI:** 10.1186/s12920-023-01702-9

**Published:** 2024-02-23

**Authors:** Hongbiao Ren, Wenhua Lv, Zhenwei Shang, Liangshuang Li, Qi Shen, Shuai Li, Zerun Song, Xiangshu Cheng, Xin Meng, Rui Chen, Ruijie Zhang

**Affiliations:** College of Bioinformatics Science and Technology, Harbin Medical University, 150086 Harbin, China

**Keywords:** IgA nephropathy, WGCNA, Immune cell infiltration analysis, Viral infection, Bacterial infection, Functional subtypes

## Abstract

**Background:**

IgA nephropathy (IgAN) is one of the most common primary glomerulonephritis, which is a significant cause of renal failure. At present, the classification of IgAN is often limited to pathology, and its molecular mechanism has not been established. Therefore we aim to identify subtypes of IgAN at the molecular level and explore the heterogeneity of subtypes in terms of immune cell infiltration, functional level.

**Methods:**

Two microarray datasets (GSE116626 and GSE115857) were downloaded from GEO. Differential expression genes (DEGs) for IgAN were screened with limma. Three unsupervised clustering algorithms (hclust, PAM, and ConsensusClusterPlus) were combined to develop a single-sample subtype random forest classifier (SSRC). Functional subtypes of IgAN were defined based on functional analysis and current IgAN findings. Then the correlation between IgAN subtypes and clinical features such as eGFR and proteinuria was evaluated by using Pearson method. Subsequently, subtype heterogeneity was verified by subtype-specific modules identification based on weighted gene co-expression network analysis(WGCNA) and immune cell infiltration analysis based on CIBERSORT algorithm.

**Results:**

We identified 102 DEGs as marker genes for IgAN and three functional subtypes namely: viral-hormonal, bacterial-immune and mixed type. We screened seventeen genes specific to viral hormonal type (ATF3, JUN and FOS etc.), and seven genes specific to bacterial immune type (LIF, C19orf51 and SLPI etc.). The subtype-specific genes showed significantly high correlation with proteinuria and eGFR. The WGCNA modules were in keeping with functions of the IgAN subtypes where the MEcyan module was specific to the viral-hormonal type and the MElightgreen module was specific to the bacterial-immune type. The results of immune cell infiltration revealed subtype-specific cell heterogeneity which included significant differences in T follicular helper cells, resting NK cells between viral-hormone type and control group; significant differences in eosinophils, monocytes, macrophages, mast cells and other cells between bacterial-immune type and control.

**Conclusion:**

In this study, we identified three functional subtypes of IgAN for the first time and specific expressed genes for each subtype. Then we constructed a subtype classifier and classify IgAN patients into specific subtypes, which may be benefit for the precise treatment of IgAN patients in future.

**Supplementary Information:**

The online version contains supplementary material available at 10.1186/s12920-023-01702-9.

## Background

### Research status of immune mechanism of IgAN

IgA nephropathy (IgAN) is the most common form of primary glomerulonephritis worldwide. It is the primary cause of chronic kidney disease, leading to progression to end-stage renal disease in 25–50% of patients within 20 years after diagnosis and reducing life expectancy by ten years. The prevalence of IgAN varies widely by race and geography [1], and the clinical manifestations of IgAN present diverse symptoms [2]. The current diagnosis of IgAN requires renal biopsy due to the lack of sufficiently specific and sensitive biomarkers. There are also several histologically classified pathological grading systems for IgAN, such as the Oxford classification of IgAN, the Lee grading system [3], and the Hass grading system [4]. In addition, the available studies suggest that its pathogenesis involves multiple factors such as immunity, genetics, environment or nutrition [5] and that the specific pathogenesis has not been fully established.

Disease-related pathological features provide clinicians with useful prognostic information and then guide treatment decisions. Besides, multiple immune cells and immune molecules in the immune system are indeed involved in the pathogenesis of IgAN through various mechanisms. For instance, secretion of cytokines such as IFN-γ, TNF-β, IL-5, IL-9 by Th1 lymphocytes and Th2 lymphocytes is involved in the pathogenesis of IgAN [5]. B cells in the early stages of IgAN pathogenesis and mucosal reactions involving APRIL and B cell activating factor BAFF play a significant role in the development of IgAN [6]. Toll-like receptors (TLRs) are thought to be mediators of renal disease and to be involved in the pathogenesis of IgAN [7]. However the immune mechanisms regarding IgAN has not well been established.

### Complex condition of pharmacotherapy for IgAN

The complexity of IgAN directly leads to complex medical therapy. Clinically, there are immunosuppressive and non-immunosuppressive therapies for patients with IgAN. Immunosuppressive steroid therapy significantly reduces proteinuria, prevents deterioration of renal function in patients with IgAN [8], and reduces mesangial matrix accumulation in advanced patients with IgAN. For non-immunosuppressive therapy, angiotensin-converting enzyme inhibitors or angiotensin II receptor blocker blockade of RAAS therapy is clinically preferred for patients with IgAN [9]. And the early anti-proteinuric effect of RAAS blockade and its local anti-inflammatory effects have been demonstrated to have significant renal benefits in both adult and pediatric populations.

To date, there is no disease-specific treatment or radical cure for IgAN patients. Whether to treat IgAN patients with angiotensin-converting enzyme inhibitors (ACEI) alone [10] or to add steroid or fish oil supplements in combination [11] depends mainly on the presence of clinical symptoms associated with poor prognosis. Furthermore, it has been shown that the addition of immunosuppressive therapy in patients with high-risk IgAN did not significantly improve outcomes, but more sided effects were observed instead. Therefore, it is necessary to classify IgAN patients from the functional level, which will be beneficial for the precision treatment of IgAN in the future.

### Overview of this study

IgAN patients have diverse clinical symptoms and high heterogeneity. The current disease grading of IgAN is mainly limited to the pathological grading level. The response to medical treatments varies, and there is a lack of diagnostic biomarkers for IgAN. Therefore, identifying molecular markers and classifying IgAN patients at functional levels may provide personalized treatments that are more beneficial to different patients. In addition, our research team previously performed a classification study of systemic lupus erythematosus (SLE) [12], which classified SLE patients into viral subtype, bacterial and fungal subtype and "mixed" subtype, and revealed the molecular mechanism of SLE subtypes for the first time based on biological molecular function. Since both SLE and IgAN belong to autoimmune disease, the previous study provides an essential foundation for our research.

In this study, we determined the global characteristics of IgAN based on two sets of IgAN transcriptome data and developed a single-sample subtype classifier. Next we constructed biofunctional co-expression networks to reveal the molecular functions of each subtype. And finally, cells related to IgAN subtypes were identified by immune cell infiltration analysis. We hope that the functional subtypes of IgAN and the biomarkers associated with the subtypes can help patients in clinical diagnosis and personalized treatment.

## Materials and methods

### Data download and processing

Gene expression data were collected from the Gene Expression Omnibus (GEO) database (http://www.ncbi.nlm.nih.gov/geo/) for patients with IgAN and healthy controls. Two datasets including GSE115857 and GSE116626 were collected in our study: for which the platform was GPL14951 (Illumina HumanHT-12 WG-DASL V4.0 R2 expression beadchip). GSE115857 contained 55 IgAN samples and 7 healthy samples, and GSE116626 contained 52 IgAN samples and 7 healthy samples. First, a probe corresponding to multiple genes in the expression profile and a probe that is not annotated to any genes are removed. Secondly, for multiple probes corresponding to the same gene, the median expression value of multiple probes is taken as the expression level of the gene. Finally, batch effects were removed using the ComBat function in the sva R package when merging the two sets of expression profiles containing 107 IgAN samples and 14 healthy controls. Since the original data has been logized, the scale function was used to normalise the data set.

### Differential expression analysis between patients with IgAN and healthy controls and functional enrichment analysis

Differentially expressed genes (DEGs) between patients with IgAN and healthy controls were identified by setting adjusted P value less than 0.05 and the absolute logarithmic fold change greater than 1.7 using the limma package. The threshold for absolute logarithmic fold change is generally between 0.5 and 2 [13, 14]. These DEGs were then visualized by volcano plots. Functional enrichment analyses for GO [15] and KEGG [16–18] were performed by ggplot2 package, enrichplot package and ClusterProfiler package.

### Construction of single-sample subtype classifier

107 IgAN samples were clustered based on expression values of DEGs between IgAN samples and healthy controls using hclust [19], PAM and consensus clustering [20] and then a category label was assigned to each IgAN sample. The optimal number of subtypes is 3 determined by the clusGap function in factoextra package, the pamk function in fpc package, and the ConsensusClusterPlus function in ConsensusClusterPlus package. A subtype label was assigned to a IgAN sample whenever any two of the three algorithms classify the sample as a same class. The whole dataset was divided into a training set and a testing set in a ratio of 3:1. A one-sample random forest classifier was constructed using the randomForest package in R based on DEGs between IgAN samples and healthy controls. By training the classifier model in the training set, we obtained the best classification model with mtry = 9 and ntree = 400 when the OOB estimate of error rate was 7.41%. In this model ntree is the number of base classifiers to be included (default is 500), and mtry is the number of variables to be included in each decision tree. In this research, the single-sample classifier achieves optimal performance with mtry = 9 and ntree = 400. Therefore mtry = 9 and ntree = 400 were chosen.The classifier model was trained with the training set, and the best model was determined with the testing set, by calculating the model prediction accuracy and the area under the curve(AUC) of receiver operator characteristic (ROC) curves since AUC is more suitable for evaluating the performance of triclassification problems than recall [21].

### Differential expression analysis between IgAN subtypes and functional enrichment analysis

To investigate the heterogenicity between different subtypes of IgAN, we identified subtype-specific expression genes using the limma package. Gene set enrichment analysis (GSEA) for each specific gene set was performed using the ClusterProfiler packages, with visualization aided by the ggplot2 and enrichplot package. Two measures including adjusted P values and absolute log-fold change were used to select subtype specific genes. As we know, inconsistent feature numbers can lead to overlapping functional pathways and affect the functional definition of the subtype. In order to balance the number of features associated with each subtype to determine the function of the subtype, different criteria were used for each subtype. Specific genes for Subtype I were identified by setting adjusted P value less than 0.01 and the absolute logarithmic fold change(|log2(FC)|) greater than 2 between subtype I and other subtypes. Specific genes for subtype II were identified by setting adjusted P value less than 0.01 and |log2(FC)| greater than 1.5 between subtype II and other subtypes. Specific genes for subtype III were identified by setting adjusted P value less than 0.01 and and |log2(FC)| greater than 1.2 between subtype III and other subtypes.

### Assessment of subtype related pathway activity

To functionally interpret the subtypes, we measured the activity of subtype related pathways by using the gene expression bias method [22]. First, we extracted the expression values of genes in the subtype-related pathways and calculated the difference between the average expression values of IgAN group and that of the normal group for each gene. Then a normalized value for a gene was obtained by dividing by the standard deviation of expression levels of the normal group. Finally the average of the absolute normalized values for genes in a certain subtype related pathway was taken as the activity score. Subsequently, we used ANOVA to assess the significance of the pathway activity scores for different subtypes.

### Correlation between subtype-specific genes and clinical features of IgAN

The intersection of DEGs between IgAN samples and healthy controls and DEGs between one subtype and the other subtypes was used as subtype-specific genes, which was then visualized using a Venn diagram plotted by the VennDiagram package and ggplot2 package in R. In addition, Pearson correlation analysis was used to calculate the correlation between subtype-specific genes and IgAN clinical features such as estimated glomerular filtration rate (eGFR) and proteinuria through the Nephroseq v5 online database (http://v5.nephroseq.org). Pearson correlation analyses were also corrected with the Benjamini-Hochberg method and p < 0.05 was considered a statistically significant correlation. The ggplot2 package in R was used for visualization.

### Construction of a co-expression network for the mixed IgAN subtype

To further investigate the linkage mechanism between the viral hormonal subtype and bacterial immune subtype, we constructed a biological process (BP) based co-expression network for the mixed subtype. The similarity of 29 biological processes associated with the viral-hormonal type and bacterial-immune type in the hybrid type was calculated using the R package rrvgo, and the associated biological processes. We then mapped the co-expression network to the mixed type using Cytoscape 3.7. Nodes in the network represent BP terms, and edges represent the magnitude of similarity.

### Construction of weighted gene co-expression network

First, 5179 genes in the top 25% with the largest variance were screened from the expression profile data of IgAN subtypes. Then the WGCNA package in R was used to construct a gene co-expression network. Subsequently, an adjacency matrix was constructed to describe the correlation degree between one node and the others. The equation of the adjacency matrix is listed as follows:

$${{\rm{p}}_{{\rm{ij}}}} = |cor({x_i},{x_j})|{a_{ij}} = {p_{ij}}^\beta $$

In this equation, x_i_ and x_j_ represent expression values of the i’th and the j’th genes respectively. p_ij_ represents the Pearson correlation coefficient and a_ij_ represents the degree of correlation between the i’th and the j’th genes.

In this study, we chose a soft threshold of β = 3 (scale-free R^2^ = 0.9). Subsequently, we transformed the adjacency matrix into a topological overlap matrix (TOM). A method to quantitatively describe the similarity of nodes by comparing the weighted correlation between two nodes and the other nodes. Next, we identified modules by hierarchical clustering where each module contained at least 30 genes (minModuleSize = 30). Co-expression networks or modules were defined as branches of a hierarchical clustering tree and each module assigned a unique color label. Finally, we computed the module feature genes, hierarchically clustered the modules, and merged those with high similarity greater than 0.75 (abline = 0.25).

### Identification of subtype-specific modules and functional enrichment analysis for modules

We identified crucial modules and hub genes associated with IgAN subtypes based on WGCNA. Co-expression modules are defined as gene collection with high topological overlap similarity, and genes in the same module usually have a higher degree of co-expression than genes in different modules. Module membership (MM) is defined as the correlation between genes and signature genes in modules and gene significance (GS) is defined as the correlation between genes and disease subtypes. These two features were applied to identify significant modules associated with IgAN subtypes. Subsequently, correlations between modules and IgAN subtypes were used to identify the specific modules associated with each IgAN subtype. Next, pathway and GO enrichment analysis of subtype modules was performed by the ClusterProfiler package where P ≤ 0.05 was considered as statistically significant. For each module, genes with |MM| > 0.7 and |GS| > 0.2 were taken as the hub genes of the module. Subsequently, modules of interest were visualized using STRING database (https://cn.string-db.org/cgi/input.pl) and Cytoscape software. Genes with interaction scores greater than 0.4 were considered significant genes.

### Immuno-infiltration analysis of subtypes

We quantified the infiltration level of 22 immune cells for each sample in the dataset by the CIBERSORT algorithm. Then we visualized the differences in immune cell infiltration between different IgAN subtypes and controls by the ggstatsplot package in R. Correlation analysis and visualization were performed using the corrplot package.

## Results

### Identification and functional characterization of DEGs in IgAN

**Fig. 1:**
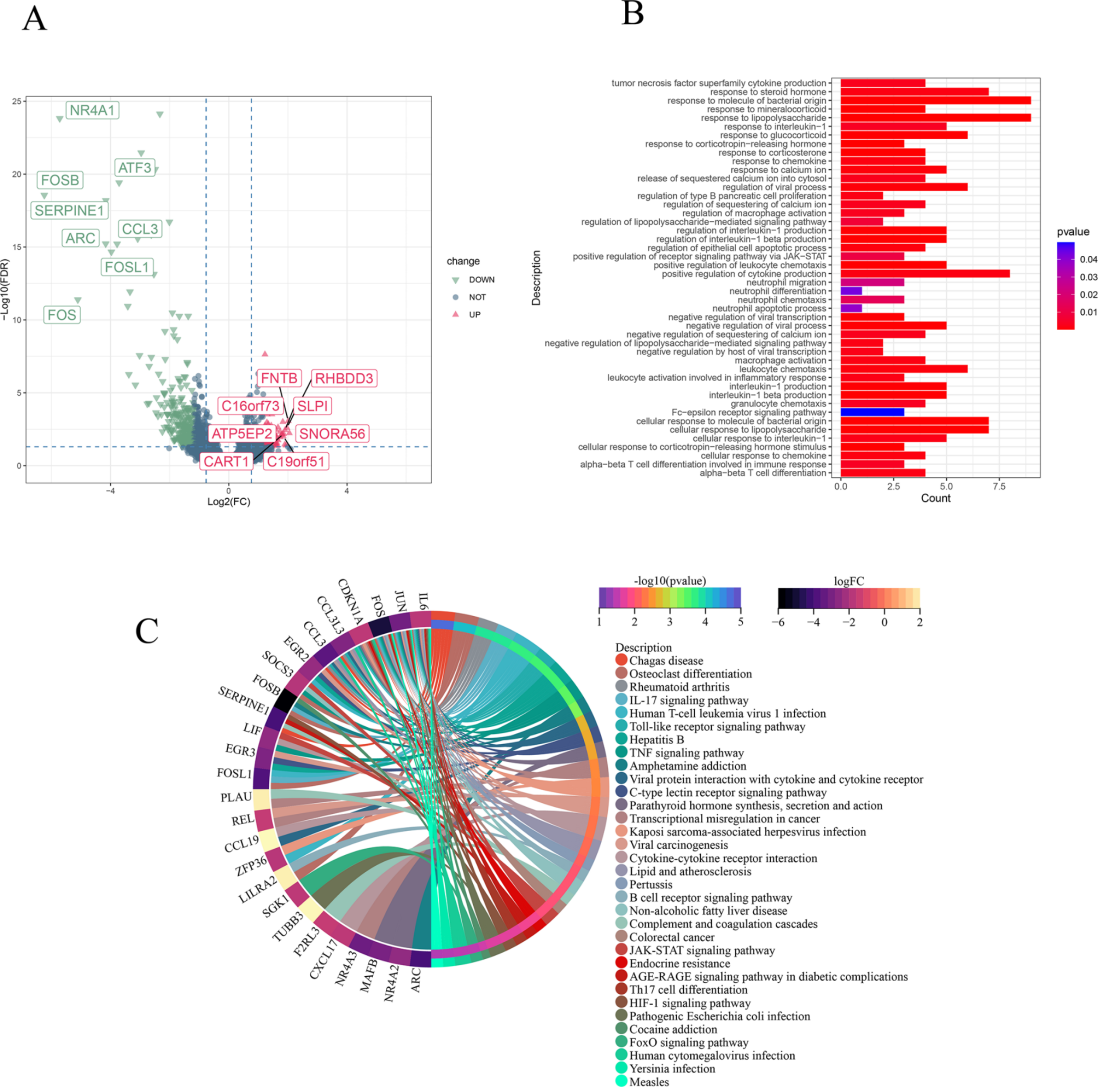
Volcano map and enrichment analysis of IgAN differential genes. **A**. Volcano map showing up-regulated and down-regulated genes in patients with IgAN. Red triangles indicate up-regulated genes, and green triangles indicate down-regulated genes. The horizontal axis represents the log2(FC) values between patients with IgAN and healthy controls. The vertical axis represents the p-value of the difference between patients with IgAN and healthy individuals. **B**. GO enrichment analysis. **C**. Pathway enrichment analysis, colors indicate the corresponding p-values and log2(FC) values.

We analyzed gene expression dataset with a total of 121 samples, including 107 patients with IgAN and 14 healthy individuals. A total of 102 DEGs (Supplementary Table 1) between IgAN patients and healthy individuals were identified, of which 13 genes were upregulated, and 89 genes were downregulated (Fig. 1A). These DEGs were significantly enriched in multiple pathways and GO terms associated with viral and bacterial infections, such as herpes simplex virus type 1 infection, coronavirus disease - COVID-19, Epstein-Barr virus infection, herpes simplex virus 1 infection, human T-cell leukemia virus 1 infection, pathogenic Escherichia coli infection, salmonella infection, epithelial cell signal transduction in Helicobacter pylori infection, and Vibrio cholerae infection (Fig. 1). Several studies [23–25] have shown that IgAN is associated with a variety of bacterial infections (Helicobacter pylori infection, Escherichia coli and Haemophilus influenzae, Staphylococcus aureus, Salmonella) and viral infections (herpes simplex virus type 1 infection, Epstein-Barr virus, and coronavirus disease-COVID-19). The results of functional enrichment analysis of DEGs in this study are consistent with previous studies on functional outcomes of IgAN. Therefore, it is reasonable to assume that DEGs and the enriched pathways are important for IgAN, and they can be used for subsequent analysis.

### Single-Sample Classifier to Predict IgAN Subtypes

We developed a single-sample random forest classifier (SSRC) to predict IgAN subtypes. The accuracy of the model calculated in the test set was 96.15%, and the AUCs corresponding to the three IgAN subtypes were 0.657, 1 and 0.976, respectively, indicating that our classifier has high classification accuracy for subtype II and subtype III. The average area under the ROC curve for the three subtypes was 0.8775 (Supplementary Fig. 1). In addition, the average accuracy of the model after 5-fold cross-validation still reaches 90.65%, further indicating that our model has a good classification efficiency. All sample labels are shown in in the Supplementary Table 2.

### Gene set enrichment analysis (GSEA) of IgAN subtypes

**Fig. 2:**
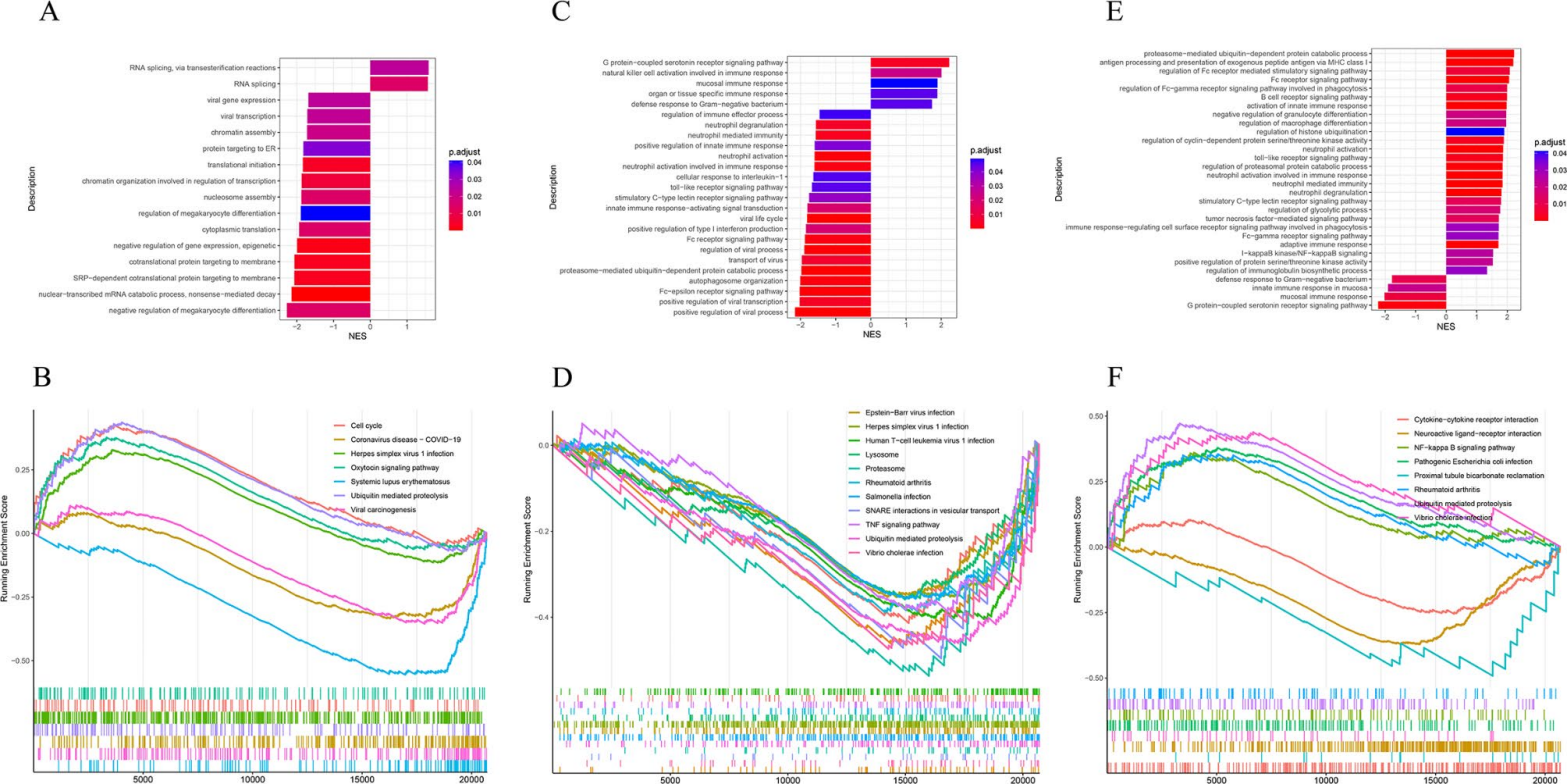
Gene set enrichment analysis of subtypes. **A**. GSEA-based GO enrichment analysis in subtype I. The horizontal axis indicates the normalized enrichment score NES, and the color indicates the corrected P value. **B**. GSEA-based pathway enrichment analysis, where the upregulated pathways in subtype I are cell cycle, ubiquitin-mediated protein hydrolysis, herpes simplex virus type 1 infection, and oxytocin signaling pathway, and the downregulated pathways are viral carcinogenesis, coronavirus disease-COVID-19, and systemic lupus erythematosus. **C**. GSEA-based GO enrichment analysis in subtype II. **D**. GSEA-based pathway enrichment analysis, in which the pathways shown are all down-regulated pathways of subtype II. **E**. GSEA-based GO enrichment analysis in subtype III. **F**. GSEA-based pathway enrichment analysis, where the upregulated pathways in subtype III are Vibrio cholerae infection, pathogenic E. coli infection, NF-kappa B signaling pathway, ubiquitin-mediated protein hydrolysis, and rheumatoid arthritis, and the down-regulated pathways were cytokine-cytokine receptor interaction, proximal tubular bicarbonate reclamation, and neuroactive ligand-receptor interaction.

The results of GSEA of IgAN subtypes revealed that subtype I was mainly enriched to viral, megakaryocyte differentiation and translated proteins terms (Fig. 2A) and virus-related pathways and protein ubiquitination pathways (Fig. 2B), such as herpes simplex virus type 1 infection, coronavirus disease - COVID-19 and ubiquitin-mediated protein hydrolysis.

Subtype II is mainly enriched to viral infection, protein ubiquitination Gram-negative bacteria, neutrophils, multiple immune-related terms(Fig. 2C) and viral infections, bacterial infections and immune pathways (Fig. 2D), such as Epstein-Barr virus infection, herpes simplex virus 1 infection, pathogenic Escherichia coli infection, Salmonella infection, epithelial cell signaling in Helicobacter pylori infection, and tumor necrosis factor signaling pathways.

Subtype III is mainly enriched to neutrophils, bacteria, macrophages and immunity terms (Fig. 2E) and bacterial infection, immune and autoimmune diseases pathways (Fig. 2F), such as defense response to gram-negative bacteria, immunoglobulin positive regulation of biosynthetic processes, B-cell receptor signaling pathway, Fc receptor signaling pathway, stimulation of C-type lectin receptor signaling pathway, Toll-like receptor signaling pathway, mucosal immune response and G protein-coupled serotonin receptor signaling pathway, Epithelial cell signaling in Helicobacter pylori infection, Pathogenic Escherichia coli infection, Salmonella infection, shigellosis, hippocampal signaling pathway, B cell receptor signaling pathway, T cell receptor signaling pathway. The detailed results of GSEA for subtypes are shown in the Supplementary Table 3.

### Patients with IgAN were defined as viral-hormonal subtype, bacterial-immune subtype and mixed subtype

**Fig. 3:**
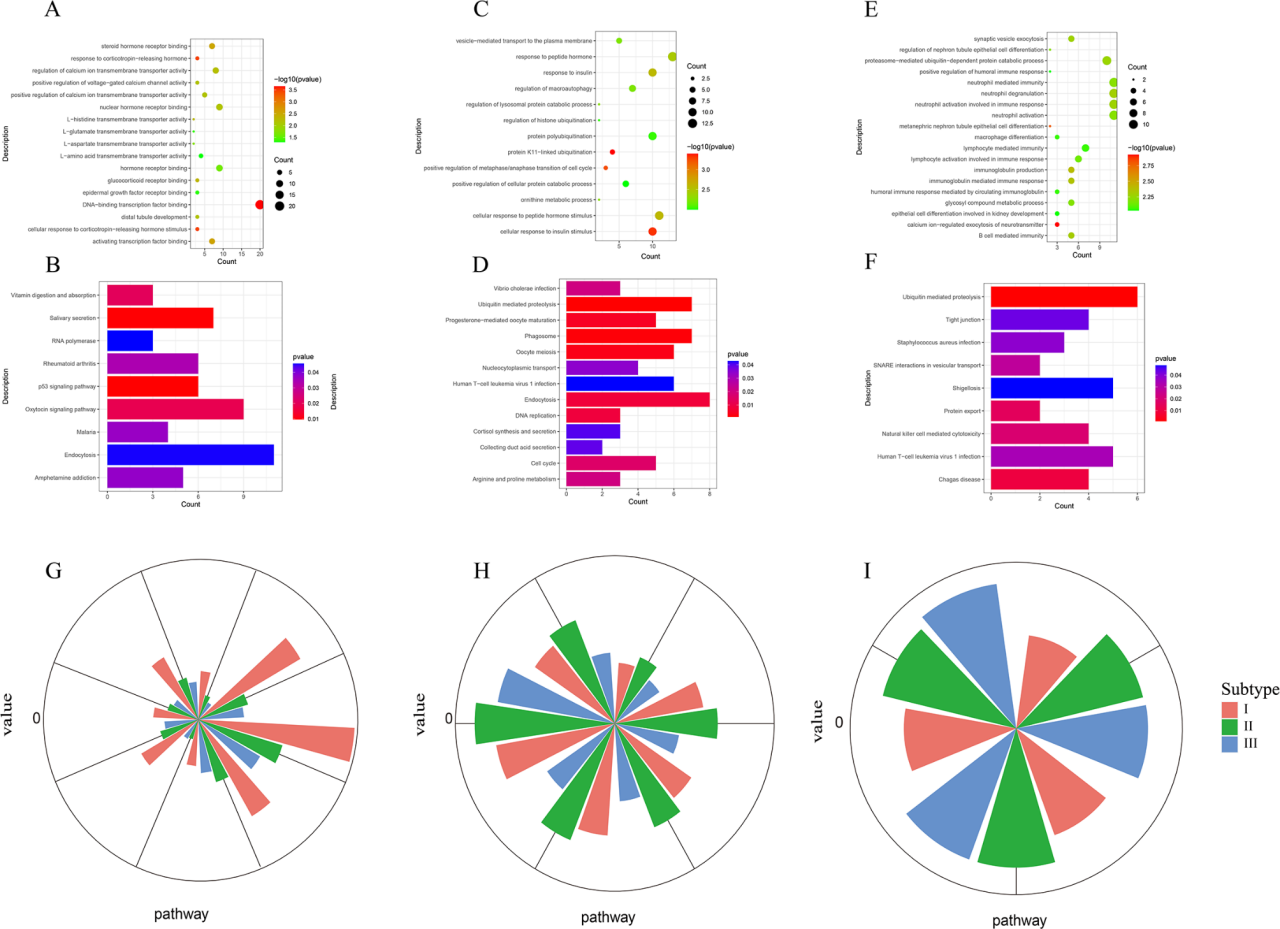
Enrichment analysis of subtypes and subtype pathway activity scores. **A-B**. Enrichment analysis of subtype I. **C-D**. Enrichment analysis of subtype II. **E-F**. Enrichment analysis of subtype III. **G, H** and **I** indicate the functional pathways associated with subtype I, subtype II, and subtype III, respectively. Each group of entries in the module represents a pathway, and the height of the bar represents the degree of dysfunction; higher pathway activity scores indicate more severe dysfunction.

580 specific genes for subtype I including 419 up-regulated genes and 161 down-regulated genes were identified. The number of specific genes for subtype II and subtype III was 250 (38 up-regulated genes 212 down-regulated genes) and 182 (169 up-regulated genes and 13 down-regulated genes) respectively. Specific genes for subtype I were mainly involved in the hormone, transcription factor and transporter protein-related GO terms (Fig. 3A) and p53 signaling pathway, oxytocin signaling pathway and rheumatoid arthritis (Fig. 3B). Specific genes for subtype II were mainly enriched in hormones, cell cycle, amino acid metabolism, and protein ubiquitination GO terms (Fig. 3C) and pathways such as ubiquitin-mediated protein hydrolysis, cell cycle, cortisol synthesis and secretion, arginine and proline metabolism, Vibrio cholerae infection and human T-cell leukemia virus type 1 infection (Fig. 3D). Specific genes for subtype III were mainly enriched in GO terms related to neutrophils, immunity, and macrophages, such as neutrophil degranulation, neutrophil activation involved in immune response, neutrophil-mediated immunity, immunoglobulin-mediated immune response, B cell-mediated immunity, regulation of renal tubular epithelial differentiation, lymphocyte-mediated immunity, macrophage differentiation and epithelial cell differentiation involved in kidney development (Fig. 3E). Specific genes for subtype III were mainly enriched in nine pathways, including ubiquitin-mediated protein hydrolysis, Chagas disease, Staphylococcus aureus infection, human T-cell leukemia virus 1 infection, natural killer cell-mediated cytotoxicity and shigellosis (Fig. 3F). The detailed results of functional enrichment analysis of subtypes are shown in the Supplementary Table 4.

Finally, we defined the functions of IgAN subtypes in combination with the results of enrichment analysis above. Subtype I is a viral-hormonal type associated with multiple viral infections, hormones, and calcium ions, and subtype III is a bacterial-immune type associated with neutrophils, various bacteria, and immunity. Subtype II is defined as a mixture of subtype I and subtype III because Subtype II contains both the multiple viral and hormone-related functions in subtype I and the neutrophil, bacterial, and immune-related functions in subtype III.

In addition, the pathway activity scores of the subtypes showed that a certain subtype-associated pathway scores were significantly higher than those of the other two subtypes (Fig. 3G-I). Meanwhile, there was a significant difference in pathway activity scores among different subtypes (p = 0.0356). Compared with scores in other subtypes, the subtype-specific pathway scores and degrees of dysregulation were highest in the corresponding subtypes, demonstrating the specificity of these pathways and heterogeneity between subtypes.

### IgAN subtype-specific genes are significantly correlated with proteinuria and eGFR

The intersection of DEGs among IgAN subtypes and 102 DEGs between all IgAN patients and healthy individuals is defined as subtype-specific genes, as shown in Supplementary Fig. 2A. We finally obtain seventeen specific genes for viral hormone subtype in which NR4A1, ATF3, FOSB, ARC, JUN, FOS, NR4A3, CCL3L3, NR4A2, ZFP36, SLC4A2, POU5F1, TSC22D4, SLC2A3, RASD1, TRIB1, and SNORA56 were up-regulated genes while SLC4A2, TSC22D4 and SNORA56 are down-regulated genes. Seven genes including LIF, SLC2A3, FOXE1, FNTB, SLPI, C19orf51 and TUBB3 specific to bacterial immune subtype are up-regulated and five genes including FOS, SLC2A3, FNTB, TRIB1, and TUBB3 are down-regulated in the mixed subtype.

Since proteinuria and eGFR are critical for IgAN, we analyzed the correlation of subtype-specific genes with these two clinical features by the Nephroseq v5 database. It has a significantly positive correlation between proteinuria and C19orf51, ATF3, NR4A1, RASD1, JUN, SLC2A3, and FOS (Supplementary Fig. 2B-H). NR4A2, TSC22D4 and LIF are positively correlated with eGFR while all the other genes (NR4A1, JUN, FOS, ZFP36, SLC4A2, RASD1, TRIB1, SLPI, TUBB3, NR4A2, TSC22D4 and LIF) are negatively correlated with eGFR (Supplementary Fig. 3A-P). In summary subtype-specific genes are highly correlated with both clinical features e.g. proteinuria and eGFR (Table 1, adjusted pvalue < 0.05), further verifying the association between subtype specific genes and IgAN. It suggests that these subtype specific genes may be involved in the development of IgAN. Therefore it is feasible for us to classify patients with IgAN into subtypes based on these genes.

**Table 1:** Subtype-specific genes and their significance with clinical features

genesymbol	subtype1	subtype2	subtype3	proteinuria	eGFR
NR4A1	+			*	*
ATF3	+			*	NA
FOSB	+			NA	NA
ARC	+			NA	NA
JUN	+			*	*
NR4A3	+			NA	NA
CCL3L3	+			NA	NA
NR4A2	+			NA	*
ZFP36	+			NA	*
SLC4A2	+			NA	*
POU5F1	+			NA	NA
TSC22D4	+			NA	*
RASD1	+			*	*
SNORA56	+			NA	NA
FOS	+	+		*	*
SLC2A3	+	+	+	*	NA
FNTB		+	+	NA	NA
TRIB1	+	+		NA	*
TUBB3		+	+	NA	*
LIF			+	NA	*
FOXE1			+	NA	NA
SLPI			+	NA	*
C19orf51			+	*	NA

Note: We use “+” to indicate that the gene is subtype-specific, “-” to suggest that the gene is not subtype-specific, “*” to indicate that the gene is significantly associated with clinical features, and "NA" to indicate that there are no corresponding clinical data.

### Linkage mechanism between the viral hormonal subtype and bacterial immune subtype demonstrated by co-expression network

**Fig. 4:**
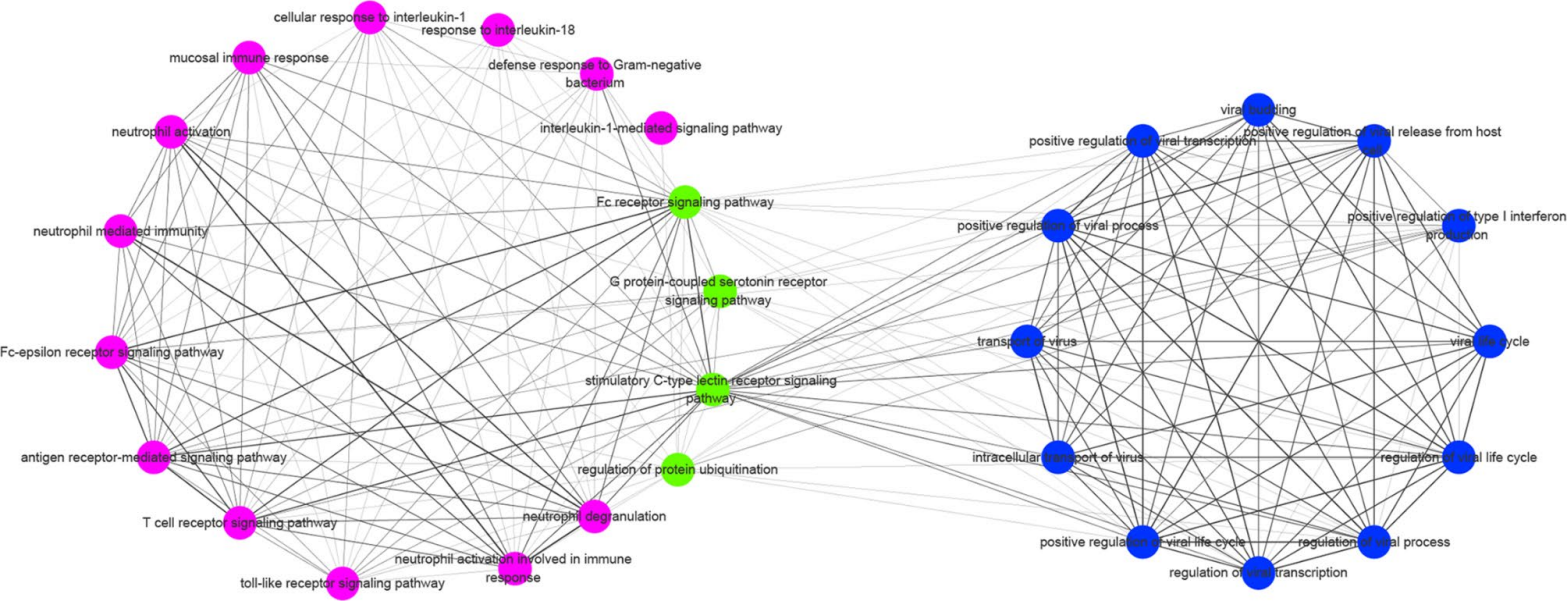
Co-expression network of the hybrid type. Each point in the network represents a BP, the edges represent the similarity between the BPs, and the edges' thickness means the similarity’s size. Points in the two subtypes are marked with different colors, and pink indicates the nodes in the mixed type associated with bacterial immunotypes, blue shows the nodes in the hybrid type associated with viral hormone types, and green nodes indicate the nodes with vital associations between the two.

To further investigate the linkage mechanism between the viral hormonal subtype and bacterial immune subtype, we constructed a biological process (BP) based co-expression network for the mixed subtype. The relevant biological processes are shown in Supplementary Table 5. It demonstrates the association of biological processes between the viral hormone type and bacterial immune type in the mixed subtype (Fig. 4), which is consistent with our expected results. Among the results, Fc receptor signaling pathway, G protein-coupled serotonin receptor signaling pathway, stimulated C-type lectin receptor signaling pathway and regulation of protein ubiquitination act as critical connecting nodes between the viral hormone subtype and bacterial immune subtype. Fc receptor and C-type lectin receptor induce phagocytosis of bacteria or fungi by activating neutrophils. Stimulation of G protein-coupled receptors leads to the release of antimicrobial protease- and peptide-containing particles, thus participating in host antimicrobial defense and inflammatory tissue damage processes.

Viruses play an essential role in activating immune cells and triggering inflammatory responses by interacting with lectins in immune cells through terminal glycans on their surfaces. Multiple C-type lectin receptors are involved in virus-induced inflammation and NETosis, and C-type lectins are involved in the current novel coronavirus pneumonia pathogenesis. Interestingly, we found that ubiquitin-mediated protein hydrolysis was significantly related to three subtypes. This finding is consistent with the fact that the regulation of protein ubiquitination act as an important node in the network and may suggest a possible role in the development of IgAN.

### Weighted Gene Co-expression Network Construction and Identification of Subtype-Specific Modules

The samples were hierarchically clustered using ward.D2 in the hclust function, and the samples were all well clustered with no outlier samples (Supplementary Fig. 4A). Subsequently, a set of candidate numbers ranging between 1 and 20 was used to filter the power values that mainly determine the scale-free properties and average connectivity of the co-expression network. Finally, in this study, we chose β = 3 as a soft threshold to construct the scale-free network, which is the minimum power value for the evaluation parameter R2 = 0.9 for scale-free networks, as shown in Supplementary Fig. 4B-E. Next, we constructed the adjacency matrix and transformed it into a topological overlap matrix (Supplementary Fig. 4G). Finally, we calculated the module eigengenes and drew the eigengene dendrogram and heatmap, as shown in Supplementary Fig. 4H. Based on hierarchical clustering and dynamic tree shearing, 33 modules were identified. Then, using 0.25 as the merging threshold, modules with a correlation higher than 0.75 are merged together to finally obtain 20 modules (Supplementary Fig. 4F), with the number of genes in the modules ranging from 40 to 1291.

**Fig. 5:**
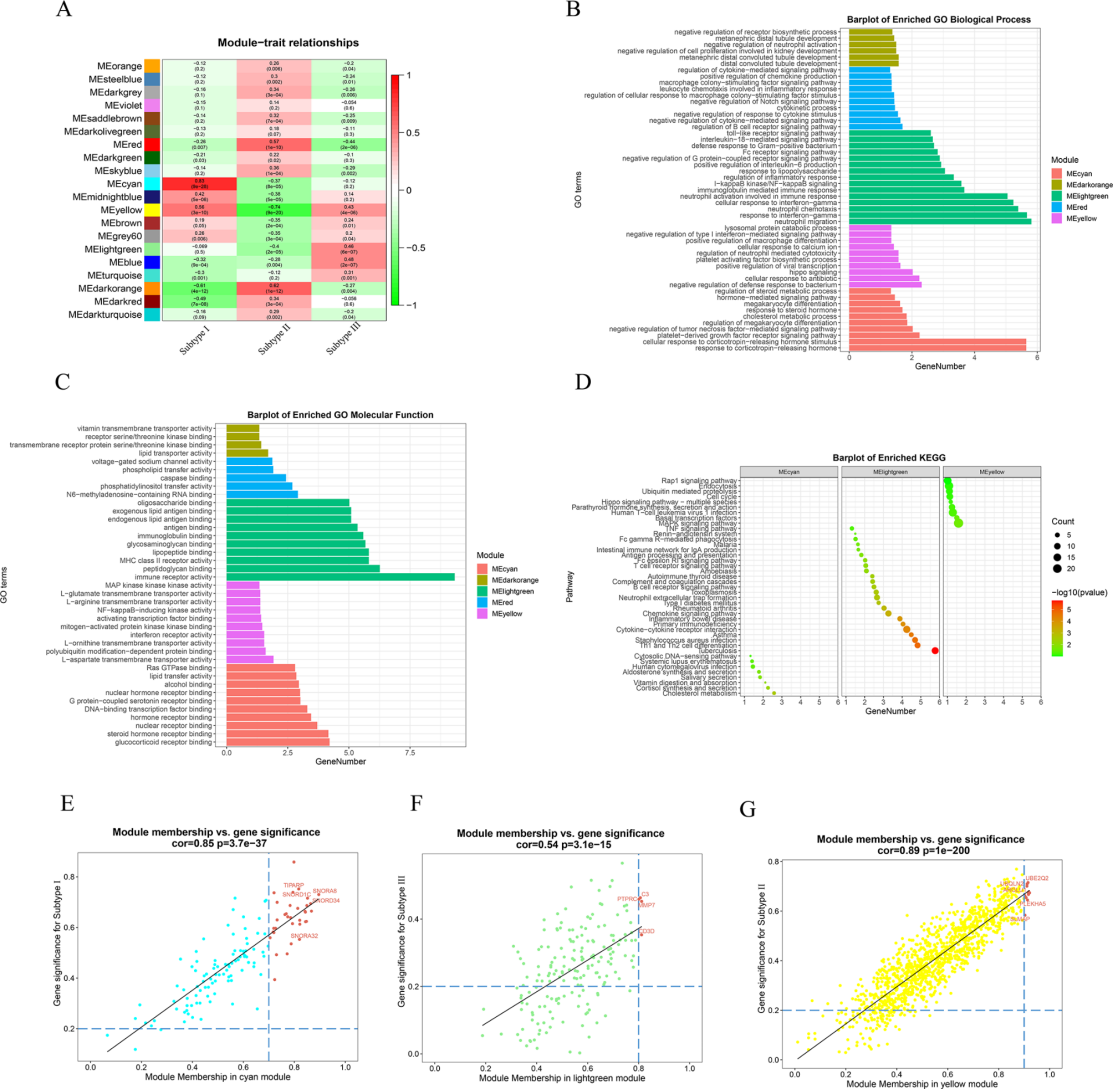
Identification of modules associated with IgAN subtypes, the functional enrichment of the module and scatter plot of the hub genes in the module. (**A**) Module-subtype correlation heat map. Rows correspond to modules, while columns correspond to subtypes, where subtype I, subtype II, and subtype III indicate viral hormonal, mixed, and bacterial immunotypes, respectively. Correlation values and P-values are shown in cells. Those in parentheses indicate P-values, and those without parentheses indicate correlation values, with red indicating a positive correlation and green indicating a negative correlation. (**B**) Biological process analysis of modules. (**C**) Molecular function analysis of modules. (**D**) Pathway enrichment analysis of cyan, light green, and yellow modules. Scatter plots of gene significance for IgAN subtype (GS) versus module members (MM) in (**E**) cyan module, (**F**) light green module, and (**G**) yellow module, correlation coefficients, and P-values are listed above the scatter plots, red indicates hub genes in each module.

The MEcyan module (coefficient = 0.83, P = 9e-28) and the MEdarkorange (coefficient=-0.61, P = 4e-12) were highly correlated with the viral hormone subtype. The MEyellow module (coefficient= -0.74, P = 9e-20) and the MEdarkorange module ( coefficient = 0.62, P = 1e-12) were highly correlated with the mixed subtype. The MElightgreen module (coefficient = 0.46, P = 6e-7) and the MEred module (coefficient=-0.44, P = 2e-6) were highly correlated with the bacterial immune subtype (Fig. 5A).These modules were selected as essential modules of IgAN subtypes for further analysis.

### Functional enrichment analysis of the modules

For the five significant modules of IgAN subtypes, we performed enrichment analysis of biological process (BP) and molecular function (MF) that all supported functional classification of the IgAN subtypes.

Specifically, the MEcyan module was mainly related to the response to corticotropin-releasing hormone, megakaryocytes, response to steroid hormones, glucocorticoid receptor binding, and G protein-coupled serotonin receptor binding. The MEyellow module was mainly related to terms such as negative regulation of bacterial defense responses, cellular response to antibiotics, hippo signaling, interferon receptor activity, and NF-kappaB-inducing kinase activity.

The MElightgreen module was mainly associated with neutrophils, interferon, interleukins, response to lipopolysaccharide, Fc receptor signaling pathway, the defense response to Gram-positive bacteria, Toll-like receptor signaling pathway, immunoglobulin binding, and antigen-binding are terms related. The MEred module was mainly related to the terms of regulation of B cell receptor signaling pathway, negative regulation of Notch signaling pathway, and voltage-gated sodium channel activity. The MEdarkorange module was mainly related to terms such as negative regulation of cell proliferation involved in kidney development, negative regulation of neutrophil activation, and receptor serine/threonine kinase binding.

In addition, we also performed pathway enrichment analysis on these significant modules. The MEcyan module was enriched in 8 pathways, including cholesterol metabolism, human cytomegalovirus infection, and systemic lupus erythematosus. The MElightgreen module was enriched in 24 pathways, including tuberculosis, S. aureus infection, complement and coagulation cascades, T cell receptor signaling pathways, and IgA production by the intestinal immune network. The MEyellow module was enriched in 9 pathways including MAPK signaling pathway, human T-cell leukemia virus type 1 infection, hippopotamus signaling pathway, and ubiquitin-mediated protein hydrolysis, etc. The results of GO and pathway enrichment analysis are shown in Fig. 5B-D, and the details are shown in the Supplementary Table 6.

### Identification of Module Hub Genes

**Fig. 6:**
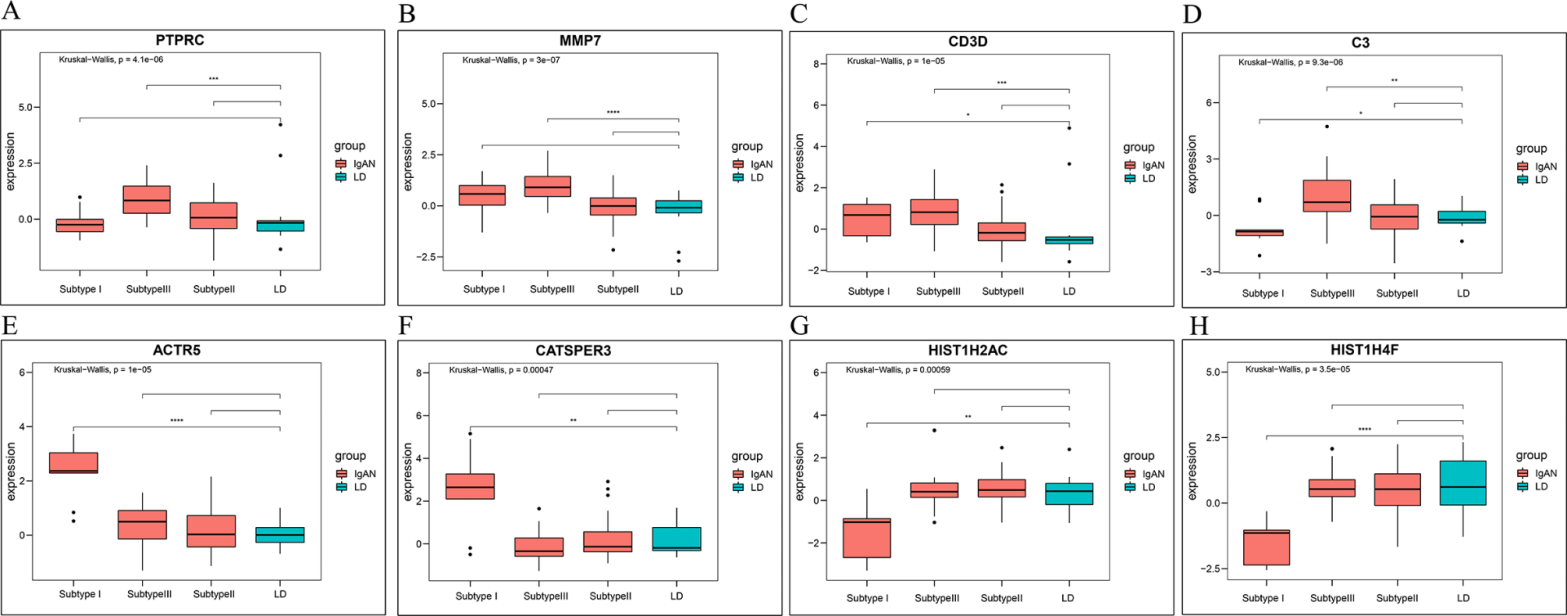
Differences in the expression levels of hub genes in the IgAN subtype and normal groups. **A-D**. Indicate the expression levels of the hub gene in light green modules. **E-H**. Indicate the expression levels of the hub gene in cyan modules in different groups. *P < 0.05; **P < 0.01; ***P < 0.001; ****P < 0.0001. Using the Wilcoxon t-test to assess the significance of differences between the two groups and the Kruskal-Wallis test to assess the overall significance of differences.

MM and GS in the significant modules were calculated, and genes in theses modules were significantly associated with the IgAN subtype (GS). They were also the essential genes in MM. Moreover, 4 hub genes (C3, CD3D, MMP7, and PTPRC) were obtained from the MElightgreen module. The number of hub genes for MEcyan module and MEyellow module is 34 (ACTR5, CATSPER3 and HIST1H2AC etc) and 13 (ARGLU1, LARP6 and PLEKHA5 etc) respectively (Fig. 5E-G). In addition, we also calculated the differences of hub genes in the and modules between IgAN subtypes and normal group. module is specific for the viral hormone subtype and MElightgreen module is specific for the bacterial immune type, consistent with the function of the modules and subtypes (Fig. 6). Besides, genes with an interaction score > 0.4 in the module and genes with an interaction score > 0.7 in the MElightgreen module were considered as significant genes. These genes mapping to the MEcyan and MElightgreen modules which were red-labeled act as the hub genes of the modules(Supplementary Fig. 5).

### Immune cell infiltration results of subtypes

**Fig. 7:**
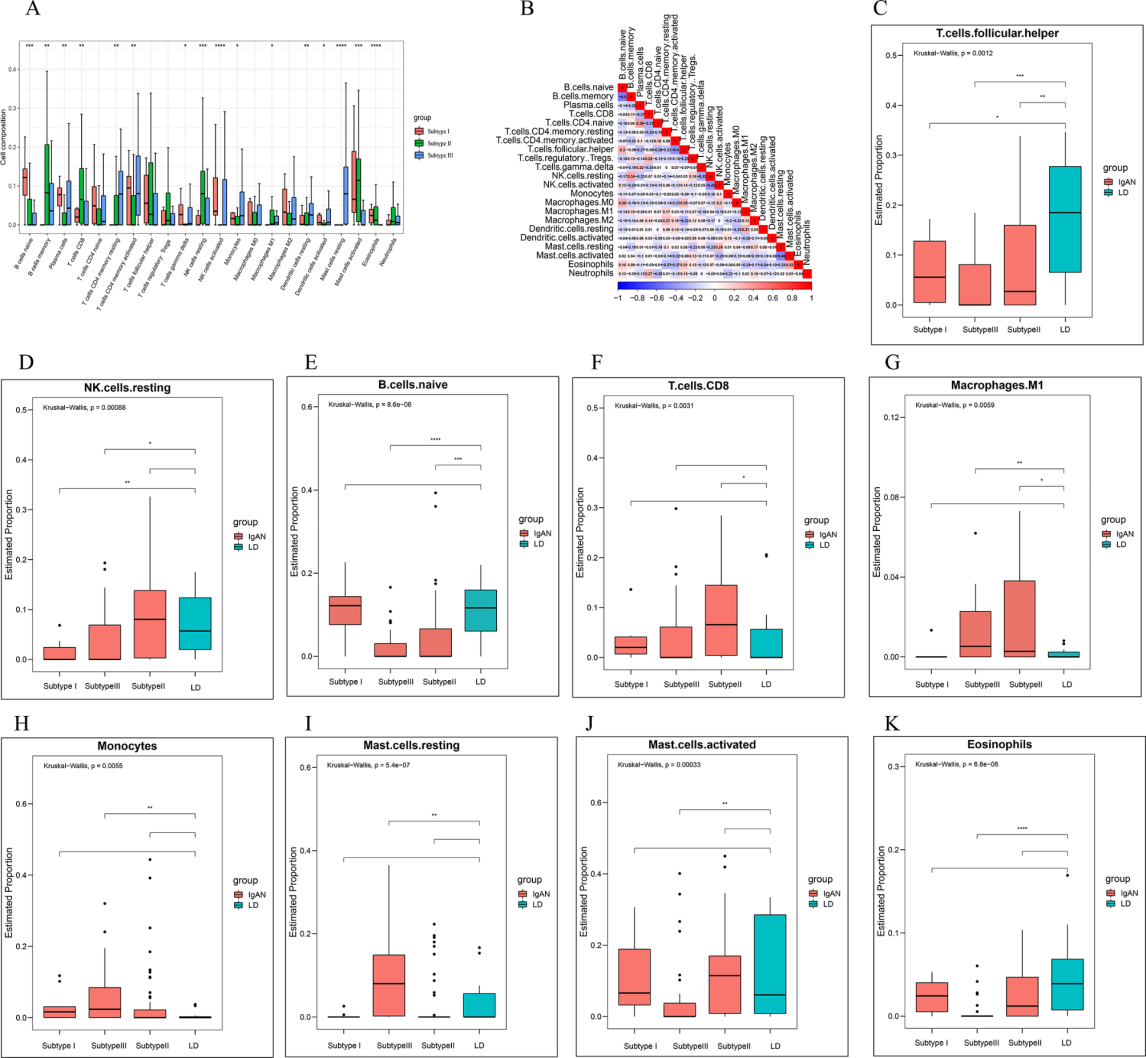
Results of immune cell infiltration of subtypes. **A**. Boxplot of the ratio of 22 immune cells estimated between IgAN subtypes. **B**. Correlation plot of 22 immune cells. Blue and red indicate negative and positive correlations, and values indicate the magnitude of the correlation. **C-D**. ​Boxplots of immune cells with significant differences between the viral hormone type and the normal group. **E-G**.​ Boxplots of immune cells with significant differences between the hybrid type and normal groups.​ **H-K**. ​Boxplots of immune cells with significant differences between the bacterial type and the normal group. Subtype I represents the hormone type of virus; Subtype II stands for mixed type; Subtype III represents bacterial immune type; *P < 0.05; **P < 0.01; ***P < 0.001; ****P < 0.0001; Using the Wilcoxon t-test to assess the significance of differences between the two groups and the Kruskal-Wallis test to assess the overall significance of differences.

We assessed the cell ratios between different IgAN subtypes based on the CIBERSORT algorithm and found significant differences in naive B cells, memory B cells, plasma cells, CD8 T cells, resting CD4 memory T cells, activated CD4 memory T cells, T cell γδ, resting NK cells, activated NK cells, monocytes, macrophages M1, resting dendritic cells, activated dendritic cells, resting mast cells, activated mast cells, and eosinophils (Fig. 7A).

In addition, we calculated the correlation between each of the two immune cell types. Naive B cells had significantly negative correlations with memory B cells (r=-0.5, P = 3.7e-9) and regulatory T cells (r=-0.18, P = 4.49e-2) and positive correlations with T follicular helper cells (r = 0.3, P = 9.89e-4) and M0 macrophages (r = 0.28, P = 1.59e-3). Memory B cells had significantly negative correlations with plasma cells (r=-0.22, P = 1.46e-2), activated CD4 memory T cells (r=-0.23, P = 9.73e-3), activated NK cells (r=-0.23, P = 1.01e-2), and resting mast cells (r=-0.19, P = 3.61e-2) and positive correlations with resting NK cells (r = 0.34, P = 1.30e-4); M1 macrophages had significantly negative correlations with eosinophils (r=-0.23, P = 1.03e-2) and activated dendritic cells (r=-0.22, P = 1.53e-2) and positive correlations with resting dendritic cells (r = 0.31, P = 5.7e-4); activated mast cells had significantly positive correlations with eosinophils (r = 0.32, P = 4.32e-4) (Fig. 7B, Supplementary Table 7).

Finally, we compared the proportion of immune cell infiltration between the IgAN subtypes and the normal group. There was significant differences in T follicular helper cells and resting NK cells (Fig. 7C-D) between viral hormone subtype and the normal group. There was substantial differences in naive B cells, resting CD4 memory T cells, activated CD4 memory T cells, monocytes, M1 macrophages, resting dendritic cells, resting mast cells, activated mast cells, and eosinophils (Fig. 7H-K) between bacterial immune subtype and the normal group. The significant differences in naive B cells, memory B cells, CD8 T cells, activated CD4 memory T cells, regulatory T cells (Tregs), activated NK cells, M1 macrophages, and activated dendritic cells (Fig. 7E-G ) was observed between the mixed subtype and normal group, where nine cells are shown in Fig. 7, the remaining cells are shown in Supplementary Fig. 6.

## Discussion

We identified three functional subtypes: viral-hormonal, bacterial-immune and mixed type. Then we screened seventeen genes specific to viral hormonal type (ATF3, JUN and FOS etc.), and seven genes specific to bacterial immune type (LIF, C19orf51 and SLPI etc.). The subtype-specific genes showed significantly high correlation with proteinuria and eGFR which indicates the reliabity of subtype classification.Some subtype-specific genes have been proved to be related to bacteria or viruses, thereby verifying the reliability of the IgAN subtype defined in this study. For example, FOS plays a role in chronic hepatitis B infection [26]; JUN is related to human T-lymphotropic virus [27], and LIF plays a role in Escherichia coli pneumonia [28]; SLPI acts as an antimicrobial protein and inhibits neutrophil extracellular trap formation [29] and is associated with Helicobacter pylori-mediated disease [30].

Subsequently, we defined the function of each subtype by functional enrichment analysis. The viral hormone type (subtype I) is associated with multiple viral infections, hormones, and calcium ions. Current studies have shown that IgAN is associated with multiple viral infections. For instance, herpes simplex virus type 1 infection in IgAN patients causes an abnormal immune response and thus may be involved in the pathogenesis of IgAN [31]. Epstein-Barr virus (EBV) in IgAN patients transform cells to secrete Gd-IgA1 and the almost complete production of IgA1 subclasses by EBV-transformed peripheral blood cells from healthy individuals in vitro suggesting that EBV may be involved in the pathogenesis of IgAN [32].

The current coronavirus disease- COVID-19, is considered a mucosal infection and has the potential to cause IgAN through this infection route [23], and COVID-19 infection exacerbates symptoms of IgAN patients [33]. This suggests the involvement of COVID-19 in the pathogenesis of IgAN. The adrenocorticotropin-releasing hormone CRH causes the release of the adrenocorticotropic hormone ACTH from the pituitary gland, which in turn induces the secretion of the glucocorticoid GC from the adrenal cortex, and corticosteroids act by binding to the glucocorticoid receptor GCR [34]. Single nucleotide polymorphisms in the glucocorticoid receptor gene NR3C1 are associated with differences in autoimmune susceptibility and steroid sensitivity and may affect IgAN treatment sensitivity and clinical outcomes [35].TLR7 can regulate the Ca2 + signaling pathway by modulating the activity of different Ca2 + channels in the cell membrane, which in turn regulates immune cell activation, cytokine production, inflammatory response, and antiviral intrinsic immune response [36]. The above results provide solid evidence for the viral hormonal phenotype of IgAN, as defined in this study.

Subtype III is a bacterial immunotype associated with neutrophils, multiple bacterial infections, and immunity, where several studies have shown the involvement of mucosal immune responses in IgA nephropathy [37–39]. Current studies have shown that IgAN is associated with various bacterial (Gram-negative and positive) infections: for example, H. pylori infection may have a pathogenic role in IgAN by causing a strong mucosal immune response and based on renal tubular damage [40]. The virulence factor of H. pylori has been shown to promote the production of Gd-IgA1 as it downregulates enzymes involved in galactosylation, and H. pylori infection is associated with elevated Gd-IgA1 levels in IgAN patients [25]. E. coli and H. influenzae subclinical infections with Haemophilus influenzae stimulate IgA production, which may be a factor in the development and progression of IgAN [41]. These provide strong evidence for our definition of the bacterial immunotype of IgAN.

This research demonstrates the association of biological processes between the viral hormone type and bacterial immune type in the mixed subtype, such as Fc receptor signaling pathway, G protein-coupled serotonin receptor signaling pathway, stimulated C-type lectin receptor signaling pathway and regulation of protein ubiquitination. These results are consistent with previous studies. Studies have shown that Neutrophils play a key role in host defence against bacterial and fungal infections, and their inappropriate activation also leads to tissue damage in autoimmune and inflammatory diseases [42]. Neutrophils express G protein-coupled receptors, Fc receptors, adhesion receptors, cytokine receptors, innate immunity receptors, and other cell surface receptors. Activation of neutrophils by Fc receptor, C-type lectin receptor induces phagocytosis of bacteria or fungi, while stimulating toll-like receptors TLRs, G protein-coupled receptors, or other intracellular pathogen molecules [43, 44]. The above process leads to stimulation of the production of superoxide and the release of antimicrobial proteases and peptide particles containing them to participate in host antimicrobial defense and inflammatory tissue damage processes [42, 45]. In addition, neutrophils have been implicated in inflammatory damage in IgAN [40] (8674239). Human neutrophils express FcαRI, a monomeric serum IgA receptor associated with FcRγ that mediates IgA-induced inflammatory processes [46, 47]. Macrophage-induced C-type lectin CLRs are immunomodulators that induce the secretion of innate cytokines and other mediators that regulate inflammation and immunity upon recognition of the corresponding ligands, and are able to promote the expression of anti-inflammatory cytokines and to counter-regulate pro-inflammatory signalling pathways [48, 49]. Similarly, C-type lectin receptors on the surface of lymphocytes are able to induce inflammation upon recognition of the ligands. Macrophage-induced C-type lectin CLRs are immunomodulators that promote the expression of anti-inflammatory cytokines and counter pro-inflammatory signaling pathways [49]. C-type lectin receptors on the surface of lymphocytes can also induce inflammation when they recognize ligands. These results show that Fc receptors, C-type lectin receptors, and G protein-coupled receptors play important roles in IgAN.

Interestingly, ubiquitin-mediated protein hydrolysis was significantly enriched in all three subtypes. It has been shown that ubiquitin-mediated protein hydrolysis is significantly enriched in several autoimmune diseases such as Alzheimer's and Parkinson's disease [50], rheumatoid arthritis [51], and type 2 diabetes [52]). Therefore we hypothesized that ubiquitin-mediated protein hydrolysis plays a vital role in the development of IgAN.

In addition, the results of immune cell infiltration in different subtypes indicated that the viral hormone subtype had different specific cells associated with the cellular immune subtype, which is consistent with previous studies related to IgAN. Specifically B cells may be involved in the production of galactose-deficient IgA1 and the antibodies in IgAN patients [53]. The decrease in naïve B cells may be due to the activation of B cells. Dendritic cells(DCs) in IgAN patientshave an impaired ability to induce IgA production in naive B cells [54]. In addition, activated mast cells are involved in tubulointerstitial inflammation and fibrosis, adrenocorticotropin-releasing hormone, which enhances mast cell activation and promotes the release of mediators NK cells induce acute kidney injury with hematuria, and T cells can induce elevated Gd-IgA1 synthesis [55].

The results in this IgAN study is consistent with our previous studies on systemic lupus erythematosus (SLE), which identified three subtypes: viral, bacterial and fungal, and mixed subtype. In the SLE research, we systematically revealed the molecular mechanism of SLE subtypes for the first time through analysis of biological functions. Viral subtype may be primarily due to overproduction of interferon caused by viral infection, leading to the formation of autoantibodies. Bacterial and fungal subtype may be primarily due to bacterial and fungal infections that stimulate neutrophilic granulocyte (NE) to produce neutrophil extracellular traps (NETs), which cause an individual autoimmune response. In future studies, we will further explore the functional typing of other autoimmune diseases, such as RA and LN.

There are several limitations to this study. First, the data do not have available follow-up information, thus making it impossible to examine the impact of the biomarker genes on the prognostic outcome. Second, although several immune cells were found to be dysregulated, and the results were consistent with previous studies, the specific functions of these immune cells and the exact mechanistic roles among the different IgAN subtypes remain unknown. Therefore, a large amount of data is needed for further clinical validation and experimental approaches is needed to validate these biomarker genes in the future.

## Conclusion

In total, we demonstrate an analytical paradigm for identifying functional subtypes of IgAN based on transcriptomic data, three machine learning algorithms and weighted gene co-expression network analysis. We identified three functional subtypes of IgAN for the first time and constructed a subtype classifier. Then we identified subtype-specific genes, subtype-specific cells, two subtype-specific modules, and dysregulated immune cells, and determined the function of each subtype. This subtype classifier can classify patients with IgAN into a specific subtype and determine subtype related biomarkers which is benefit for the precise treatment of IgAN patients. This study proved that IgAN subtypes are heterogeneous in gene expression, functional level, and immune cells, which can be used for clinical experimentalists to carry out drug experiments according to subtypes, develop related drugs for IgAN subtype, improve the efficacy of current medical therapy.

### Electronic Supplementary Material

Below is the link to the electronic supplementary material

Supplementary Material 1

Supplementary Material 2

Supplementary Material 3

Supplementary Material 4

Supplementary Material 5

Supplementary Material 6

Supplementary Material 7

Supplementary Material 8

## Data Availability

The datasets employed in our study can be acquired in the GEO repository (https://www.ncbi.nlm.nih.gov/geo/). The accession numbers are GSE115857, and GSE116626.

## Abbreviations

IgAN: IgA nephropathy
DEGs: Differentially expressed genes
SSRC: Single-sample subtype random forest classifier
WGCNA: Weighted gene co-expression network analysis
TLRs: Toll-like receptors
ACEI: Angiotensin-converting enzyme inhibitors
SLE: Systemic lupus erythematosus
GEO: Gene Expression Omnibus
GO: Gene Ontology
BP: Biological process
MF: Molecular function
TOM: Topological overlap matrix
MM: Module membership
GS: Gene significance
GSEA: Gene set enrichment analysis
AUC: Area under the curve
ROC: Receiver operator characteristic
eGFR: Estimated glomerular filtration rate
EBV: Epstein-Barr virus
Gd-IgA1: Galactose-deficient IgA1
Tregs: Regulatory T cells
DCs: Dendritic cells
