# Supplementary material for: Identifying functional subtypes of IgA nephropathy based on three machine learning algorithms and WGCNA
Source: BMC Med Genomics. 2024 Feb 23;17:61. doi: 10.1186/s12920-023-01702-9 (PMC10893719; doi:10.1186/s12920-023-01702-9)
Supplement: Supplementary file 8 — Supplementary Material 8 [file 12920_2023_1702_MOESM8_ESM.doc]

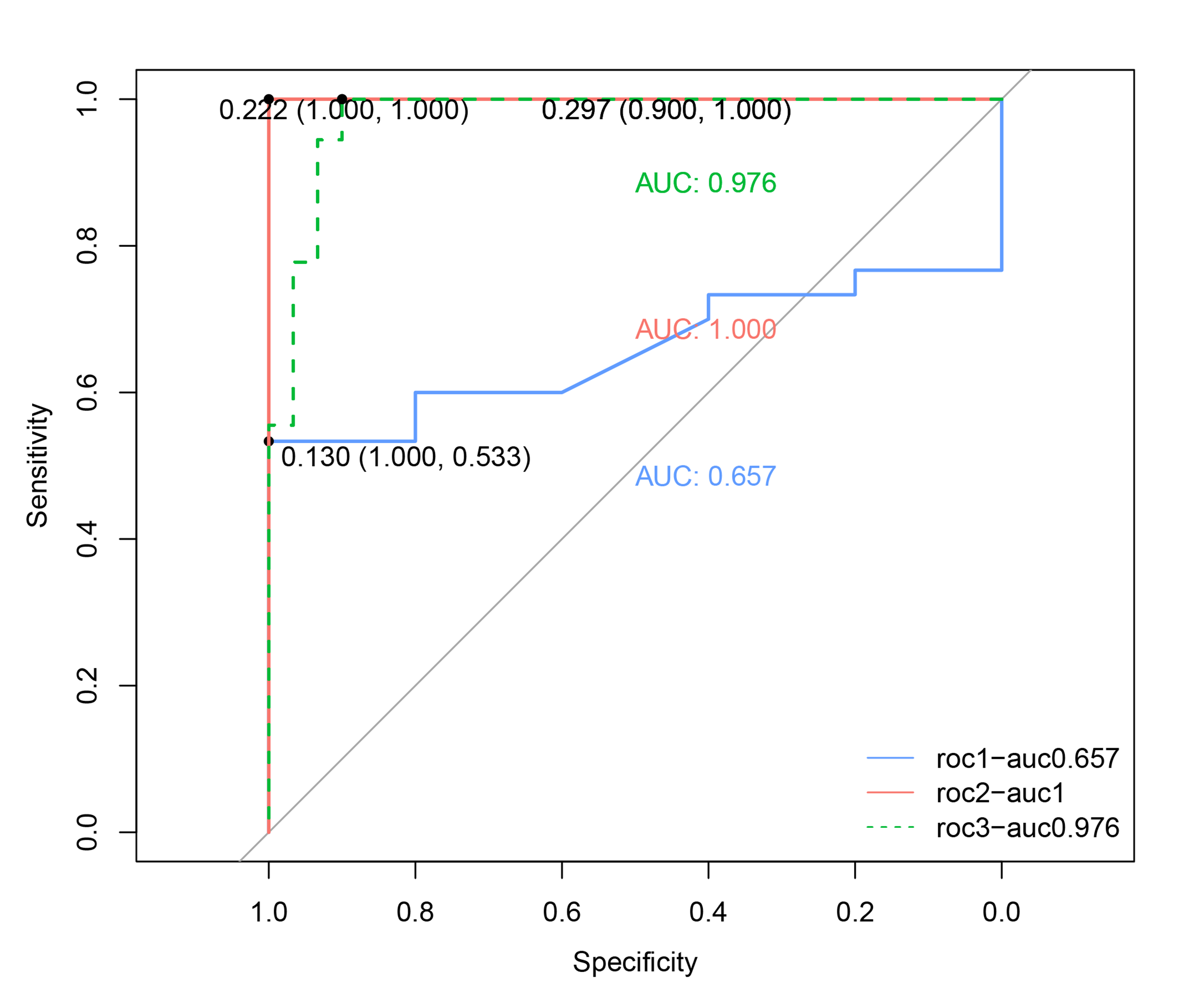


Supplementary Figure 1.ROC curves of the single-sample subtype classifier. The blue line indicates the ROC curve of subtype I, whose corresponding area under the ROC curve AUC is 0.657, where 0.130 is the best threshold point; the red line indicates the ROC curve of subtype II, whose corresponding area under the ROC curve is 1, where 0.222 is the best threshold point; the green line indicates the ROC curve of subtype III, whose related area under the ROC curve is 0.976, where 0.297 is the optimal threshold point.


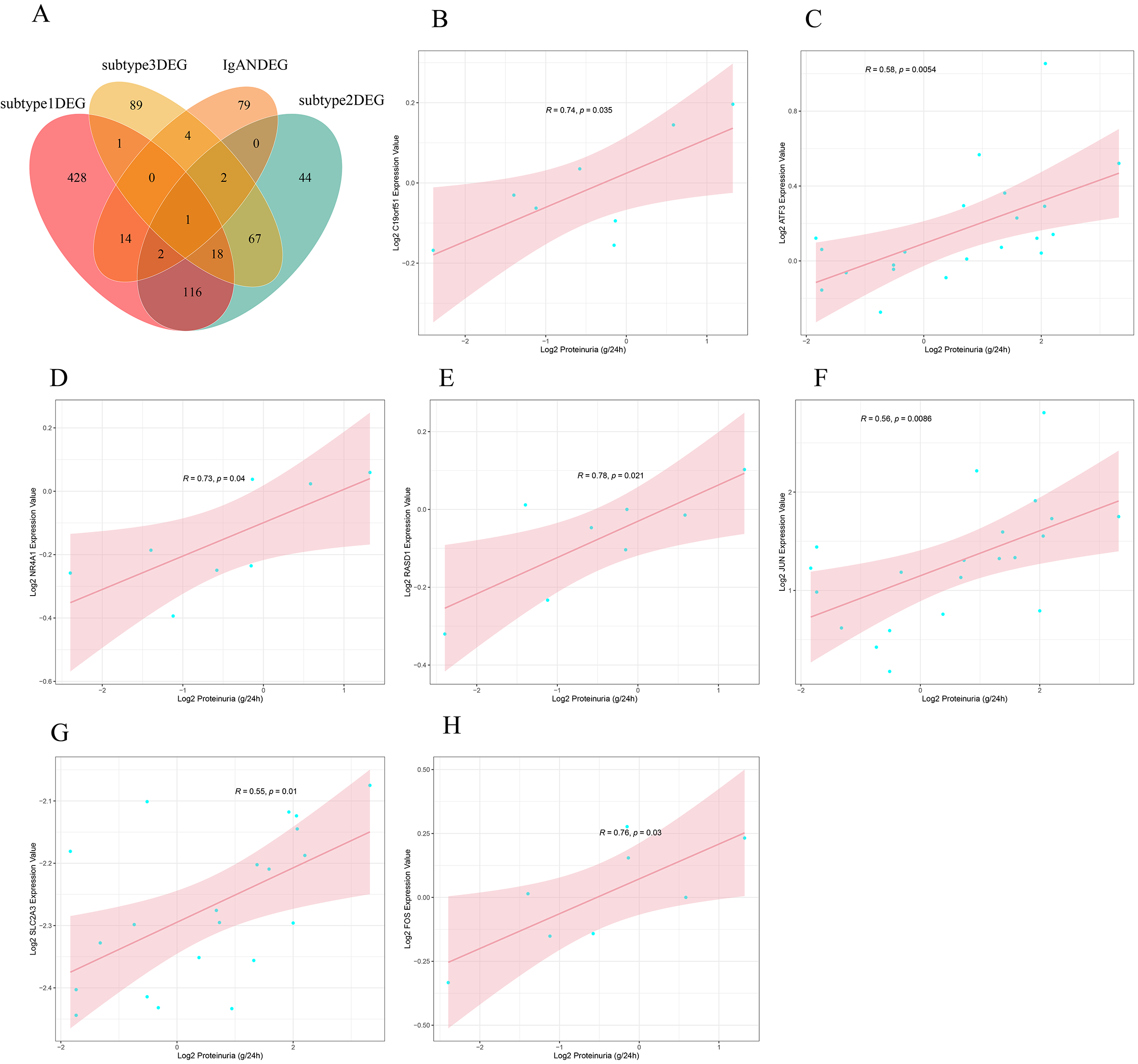


Supplementary Figure 2.Venn diagram of subtype-specific genes and their correlation with proteinuria. A. Venn diagram of subtype-to-subtype DEGs intersected with 102 IgAN DEGs. B-H. Indicates the correlation of 6 subtype-specific genes with clinical features proteinuria, containing C19orf51, ATF3, NR4A1, RASD1, JUN, SLC2A3, and FOS.


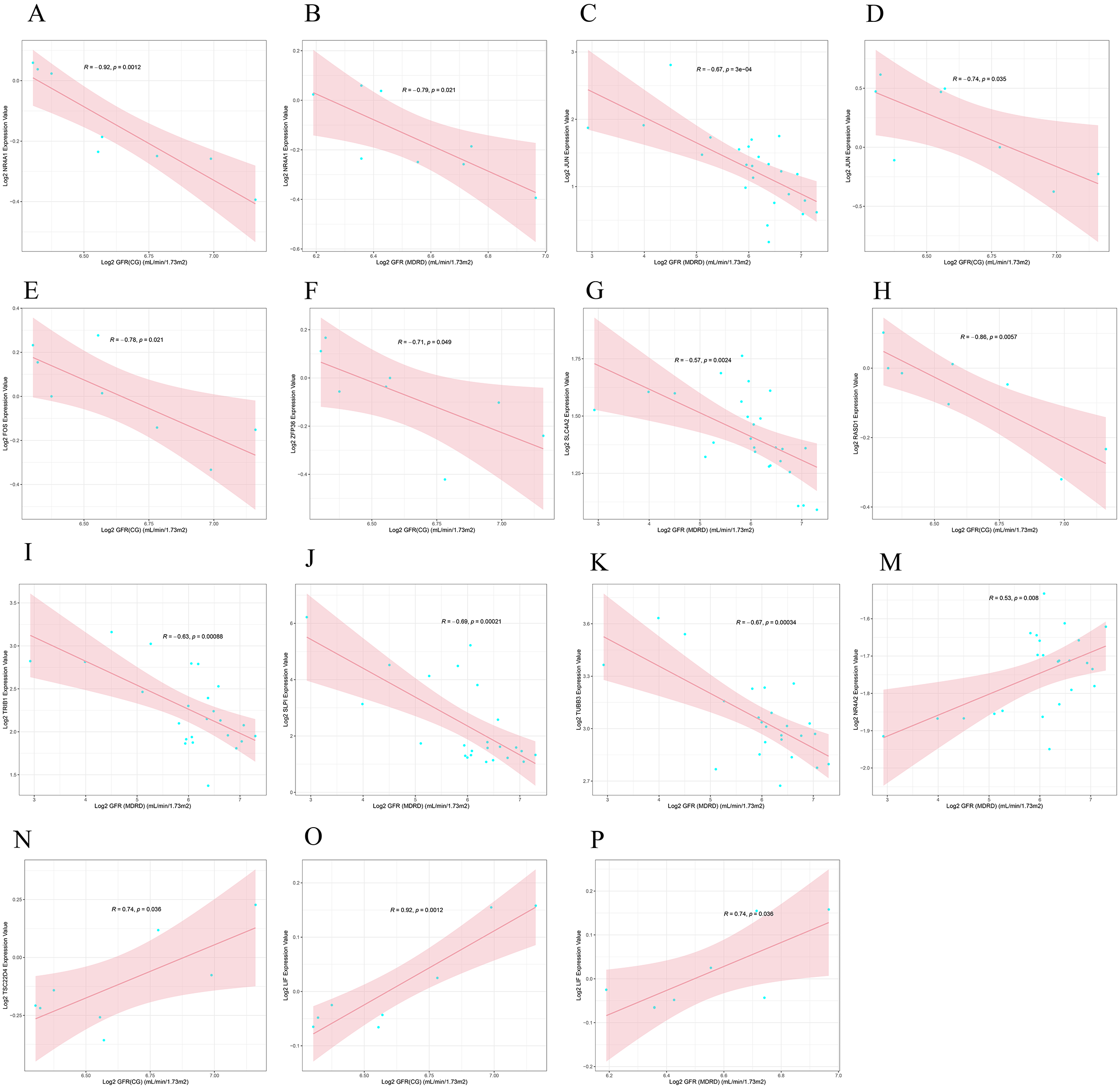


Supplementary Figure 3.Correlation between subtype-specific genes and eGFR. A-P. Indicates the correlation between 12 subtype-specific genes and clinical features eGFR, including NR4A1, JUN, FOS, ZFP36, SLC4A2, RASD1, TRIB1, SLPI, TUBB3, NR4A2, TSC22D4, and LIF.


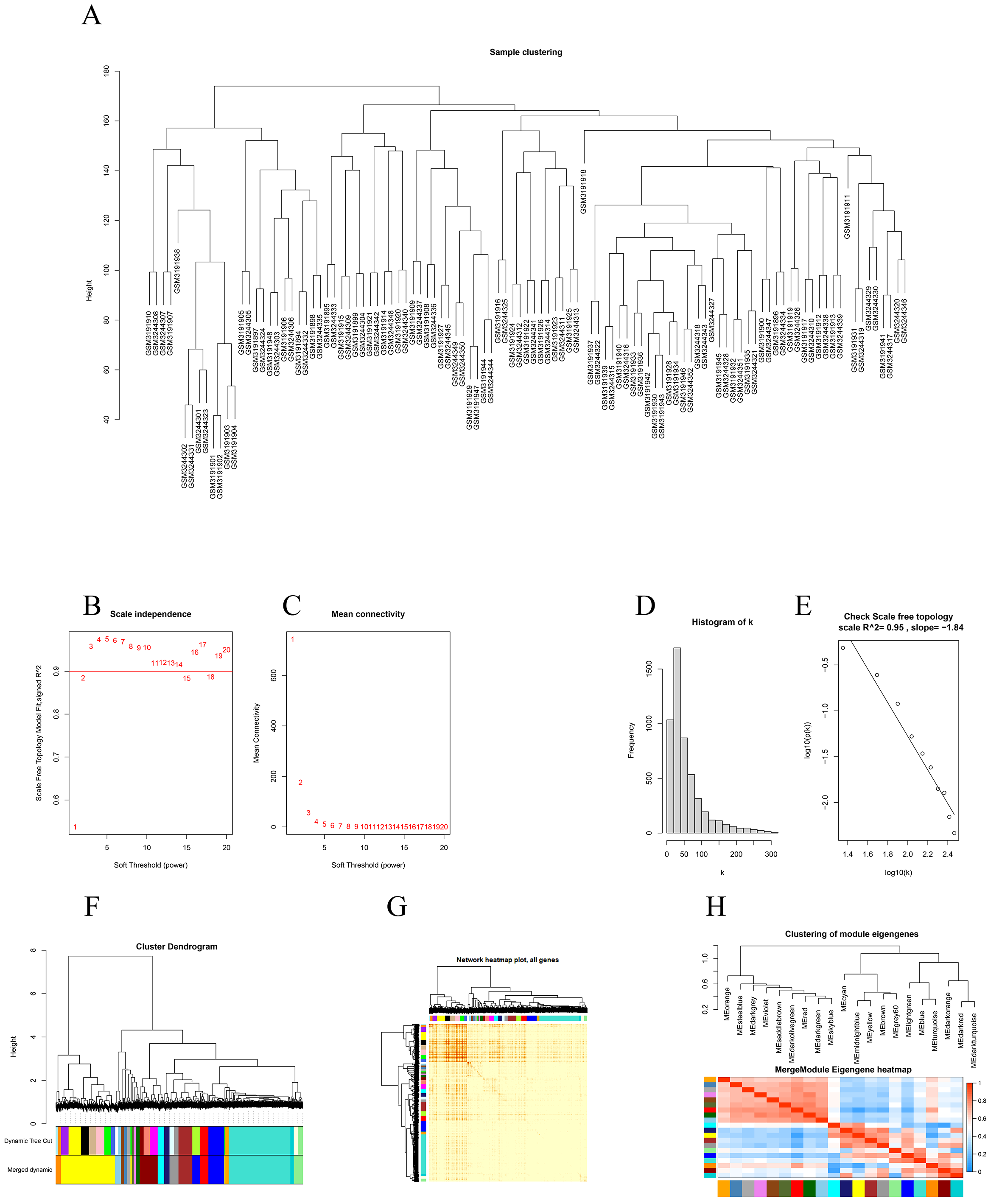


Supplementary Figure 4. Hierarchical clustering of samples in WGCNA and determination of soft thresholds and Identification of modules associated with IgAN subtypes. (A) Hierarchical clustering results for 107 samples. (B) Scale-free exponential analysis of various soft threshold powers (β). (C) Analysis of the average connectivity of various soft threshold powers. (D and E) Examination of the scale-free topology for β = 3. The x-axis shows the logarithm of the overall network connectivity, while the y-axis shows the logarithm of the corresponding frequency distribution. (F) Tree diagram of all differentially expressed genes based on differential metric (1-TOM) aggregation and assigned module colors, original (33 modules), and merged modules (20 modules) are shown in the two colored bars below, respectively. (G) Heat map of all genes. The light-to-dark red color indicates a low to high correlation. The gene tree and module assignments are shown on the left and top of the graph. (H) Clustering tree and heat map of the module feature genes. The top of the figure shows the clustering tree of modules, and the bottom of the figure shows the correlation heat map of modules, where the red to blue square colors indicate the correlation of the corresponding modules from high to low.


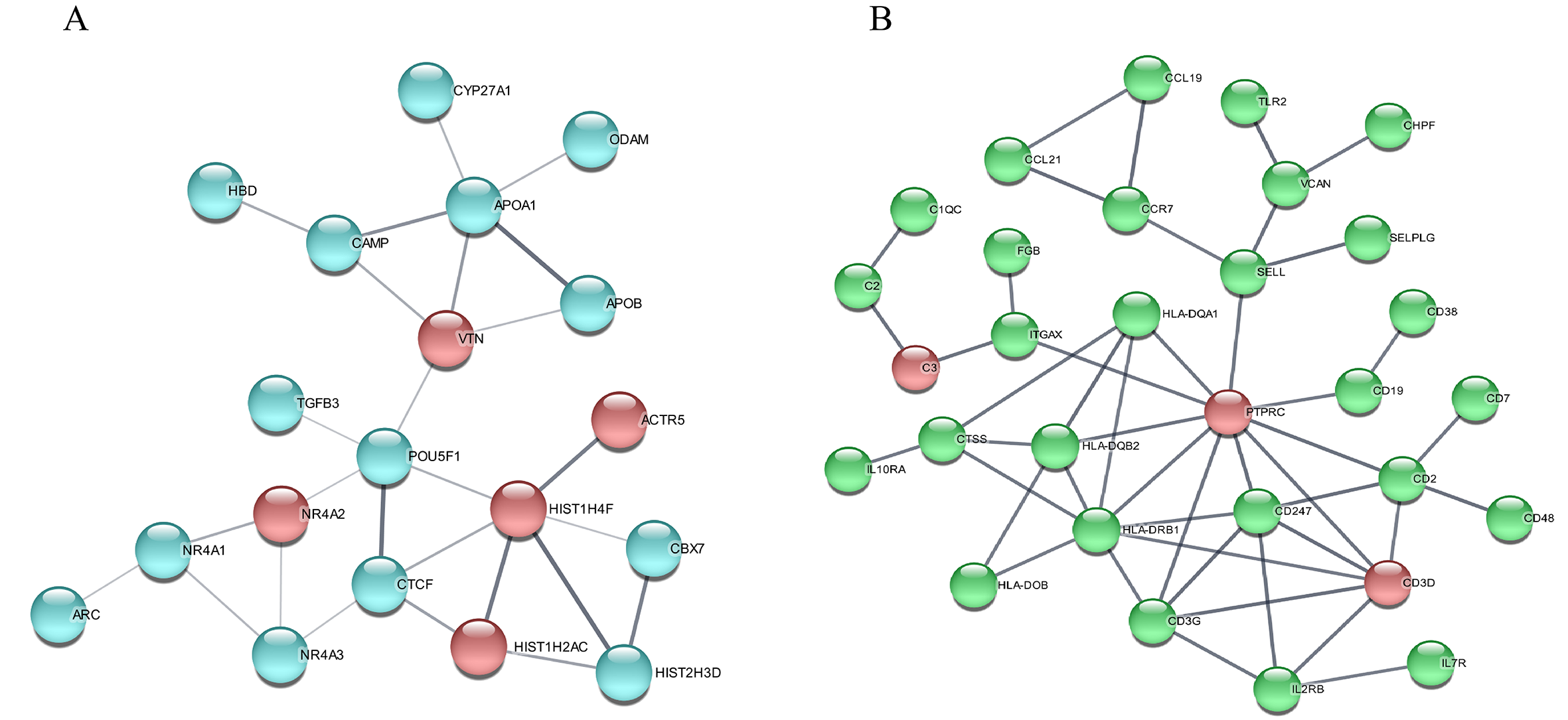


Supplementary Figure 5.Visualization of co-expression of genes in the co-expression module. A. Indicates the visualization of co-expressed genes in cyan modules. B. Indicates the visualization of co-expressed genes in light green modules. All red colors indicate the hub genes in the modules.


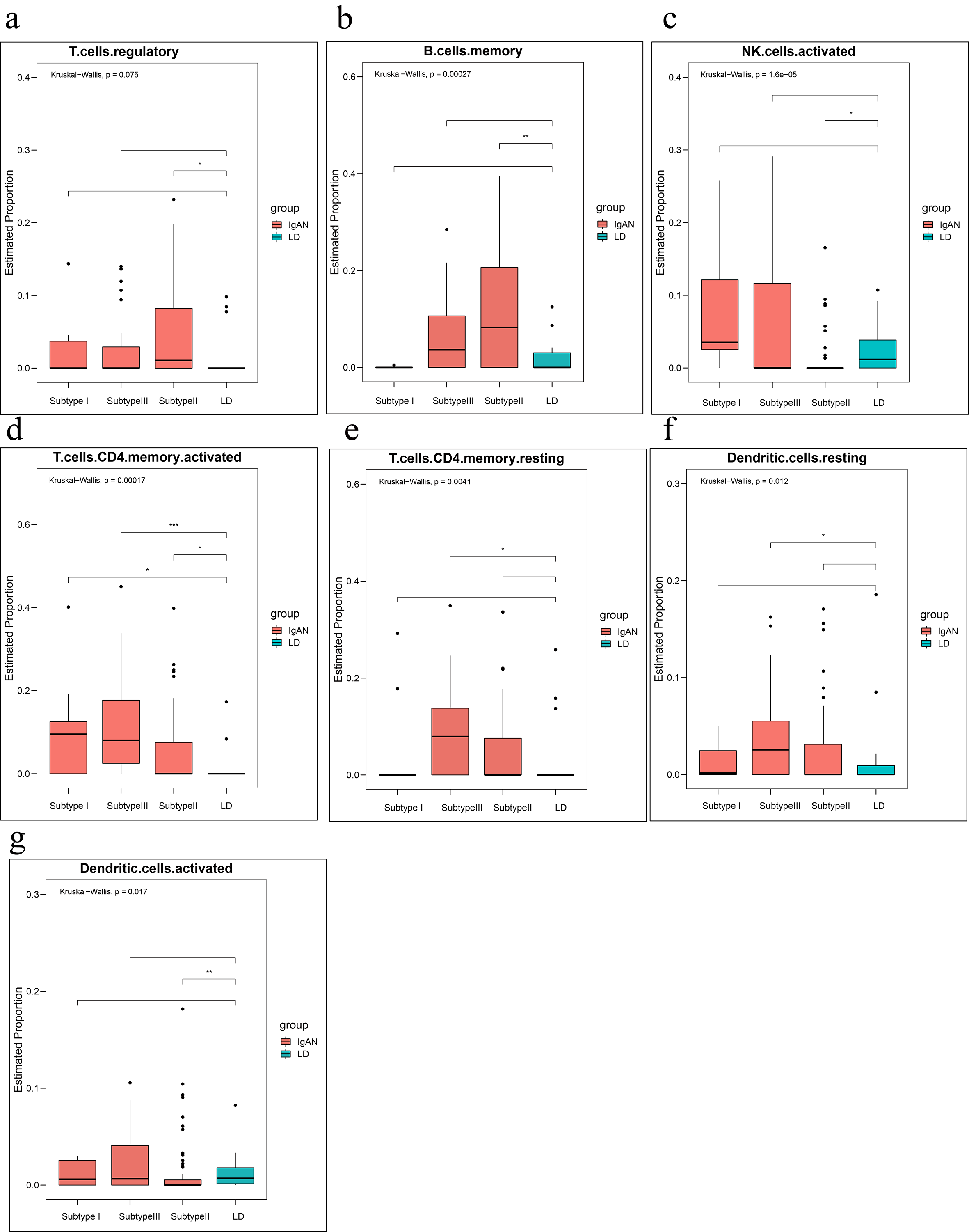


Supplementary Figure 6. Analysis of proportionally infiltrated immune cells. C1 represents the hormone type of virus; C2 stands for mixed type; C3 represents bacterial immune type; * P < 0.05; * * P < 0.01; * * * P < 0.001; * * * * P < 0.0001; Wilcoxon T test was used to assess the statistical significance of differences between pairs, and Kruskal-Wallis test was used to assess the overall significance of differences.
